# Epitope Prediction Based on Random Peptide Library Screening: Benchmark Dataset and Prediction Tools Evaluation

**DOI:** 10.3390/molecules16064971

**Published:** 2011-06-16

**Authors:** Pingping Sun, Wenhan Chen, Yanxin Huang, Hongyan Wang, Zhiqiang Ma, Yinghua Lv

**Affiliations:** 1 Faculty of Chemistry, Northeast Normal University, Changchun 130024, China; Email: sunpp567@nenu.edu.cn; 2 School of Computer Science and Information Technology, Northeast Normal University, Changchun 130117, China; Email: chenwh795@nenu.edu.cn; 3 National Engineering Laboratory for Druggable Gene and Protein Screening, Northeast Normal University, Changchun 130024, China; Email: wanghy668@nenu.edu.cn

**Keywords:** epitope prediction, mimotope, template-target complex, benchmark dataset, evaluation

## Abstract

Epitope prediction based on random peptide library screening has become a focus as a promising method in immunoinformatics research. Some novel software and web-based servers have been proposed in recent years and have succeeded in given test cases. However, since the number of available mimotopes with the relevant structure of template-target complex is limited, a systematic evaluation of these methods is still absent. In this study, a new benchmark dataset was defined. Using this benchmark dataset and a representative dataset, five examples of the most popular epitope prediction software products which are based on random peptide library screening have been evaluated. Using the benchmark dataset, in no method did performance exceed a 0.42 precision and 0.37 sensitivity, and the MCC scores suggest that the epitope prediction results of these software programs are greater than random prediction about 0.09–0.13; while using the representative dataset, most of the values of these performance measures are slightly improved, but the overall performance is still not satisfactory. Many test cases in the benchmark dataset cannot be applied to these pieces of software due to software limitations. Moreover chances are that these software products are overfitted to the small dataset and will fail in other cases. Therefore finding the correlation between mimotopes and genuine epitope residues is still far from resolved and much larger dataset for mimotope-based epitope prediction is desirable.

## 1. Introduction

A B-cell epitope is defined as a part of protein antigen recognized by either a particular B cell receptor (BCR) or a particular antibody molecule of the immune system [1]. It may be either a short contiguous stretch of amino acids, called a linear or continuous epitope, or consist of sequence segments that are brought together in spatial proximity when the protein is folded, called a conformational or discontinuous epitope [2]. It has been suggested that more than 90% of B-cell epitopes are conformational epitopes [2,3]. 

The identification of B-cell epitopes is important to many immunodetection and immunotherapeutic applications because they elicit humoral immune responses [4,5]. The objective of epitope prediction is to design a molecule that can mimic the structure and function of a genuine epitope and replace it in medical diagnostics and therapeutics, and also in vaccine design [1,6]. The most reliable methods of mapping epitopes are by X-ray crystallography or Nuclear Magnetic Resonance (NMR) [7,8,9]; however these techniques are demanding and time-consuming. Comparatively the computational methods to detect epitopes are much more efficient and cheap, but they still cannot achieve as satisfactory results as experimental methods [10]. Thus, the combination of experimental and computational approaches seems to be the best choice.

In a very long time, the computational methods for B-cell epitope prediction have focused mainly on linear epitopes. These methods are based on various amino acid propensity scales, such as hydrophilcity scale, β turns, solvent accessibility, *etc.* [11,12,13,14,15,16,17], but in 2005 Blythe and Flower confirmed “single-scale amino acid propensity profiles cannot be used to predict epitope location reliably” by the combination of propensity scales and feature parameters obtained amount to above 10^6^ experiments [18]. Later, some methods that based on machine learning and artificial intelligence were proposed [19,20,21], but in 2007 Greenbaum *et al*. demonstrated that “the combination of scales and experimentation with several machine learning algorithms showed little improvement over single scale-based methods” [22]. The 3D structure of protein can give more information than amino acid sequence, thus epitope prediction based on 3D structure of antigen will get better results. Methods that had been proposed to detect linear epitopes based on the 3D structure of protein before the first Antigen-Antibody (Ag-Ab) was solved confirmed this point [11,23,24,25,26]. With the availability of more Ag-Ab crystal complexes, epitope prediction entered a new era, and many algorithms for conformational rather than linear epitopes identification were proposed based on this significant information [27,28,29,30]. 

Since phage display technology was first propounded by Smith in 1985 as a systematic method for presenting, selecting and evolving proteins and peptides displays on the surface of filamentous phage [31], it has developed rapidly both in basic research such as the identification of protein-protein interactions [32,33], and in applied research such as the development of new diagnostics, therapeutics and vaccine design [4,34,35]. There are usually two steps in epitope prediction based on phage display technology. The first is the determination of mimotopes. The random peptides which bind to a monoclonal antibody with certain degree of affinity are screened, eluted and later amplified. According to 3–5 rounds of “adsorption-elution-amplification”, the resulting peptides become fewer but with higher affinity. These affinity-selected peptides are defined as mimotopes. They not only have high sequential similarity with the native epitope but also can mimic the essential features of the genuine epitope [36,37]. The second step is mapping the mimotopes to the antigen. In recent years, several tools have been developed to implement this step [27,28,38,39,40,41,42,43,44,45,46], and the mapping algorithms can be mainly classified into two categories according to their dependency on antigen structures: the one works with the sequence of antigen and the other works with both the sequence and the 3D structure of antigen. But no matter which way they work, the algorithms can predict both linear and conformational epitopes. 

Epitope prediction method based on random peptide library screening is supposed to improve tremendously the accuracy of the epitope prediction by combining biology experiments with computational methods [40,41,42]. Though all the existing methods have succeeded in the given test cases, a systematic evaluation of these methods is still absent because of the scarcity of useful test data [38]. Moreover, since the number of available mimotopes with corresponding structure of template-target complex is limited, the existing software is prone to be overfitted. Constructing benchmark datasets will help establish the standard for the evaluation and comparison to the existing algorithms and it is badly needed for the development of new epitope prediction algorithms. In this paper, we collected a group of reliable datasets from MimoDB [47] and PDB [48] as a benchmark dataset to facilitate the further development of this standard. In addition, using the new benchmark dataset and a representative dataset, we evaluated five recently developed epitope prediction tools: Mapitope [40], PepSurf [41], Pep-3D-Search [42], Pepitope (short for the combination method of Mapitope and PepSurf, for a detailed description see the Material and Methods section) [43] and EpiSearch [44]. All these tools are either Open Source or have freely available web services.

## 2. Results and Discussion

### 2.1. Datasets Compilation

Sixty two test sets had been collected from the MimoDB of November 2010, and the corresponding structures of template-target complexes had been obtained from the PDB. Every test set was carefully manually checked. If a test set has several corresponding complex structures in the PDB, we selected the one with the latest released date and the highest resolution (there are 24 such sets among the 62). For the sets whose whole information is the same but the round of biopanning, the last round one is retained while the others are excluded. There are 14 such sets [MS01153, MS01101, MS01102, MS01103, MS01104, MS01106, MS01107, MS01108, MS01109, MS01111, MS01112, MS01113, MS01114 and MS01153]. If sets have all the same data, they were combined as one independent set, and the mimotope sequences of these sets were combined. The MS01105, MS01110 and MS01115 meet this condition, and we combined them as MS01105/10/15. If a template-target complex structure has several pairs of template and target chains with the same structure and binding sites, only one pair was selected and the template chain was extracted. The genuine epitopes of these mimotopes sets were determined by CED, IEDB-3D or CMA. In this way, 47 test sets with 28 corresponding complex structures formed the final benchmark dataset. These 47 test sets contain 18 sets with the antigen-antibody complex and 29 sets with the protein-protein interactions. Then for the same template-target complex, we selected only one test set according to the prediction results as the representative dataset (see Material and Methods section). Finally, 30 test sets formed the representative dataset.

### 2.2. Performance Evaluation for the Existing Epitope Prediction Software

We consulted and analyzed 11 recently-developed methods (see Table 1), and finally we chose five publicly available ones to test using the benchmark dataset. The detail information of the five methods is provided in the Material and Methods section. 

**Table 1:** Comparison of 11 epitope prediction methods. A brief introduction was given.

Method	Publication year	Language	Operating System	Service	Notes
FINDMAP	2003	C++	not stated	no	FINDMAP is a method to acquire information on the 3D structure of the protein by identifying discontinuous epitopes; it maps one mimotope sequence to the protein at a time.
SiteLight	2005	C++	Linux	no	SiteLight is a method of predicting the binding site on a 3D structure using random peptide library screening.
3DEX	2005	VB	Windows	no	3DEX allows the analysis of single amino acid of a linear peptide sequence with regard to their spatial neighborhood in the 3D structures of PDB files based on preselectable parameters like distance, string length (frame size) and surface exposure. It maps mimotopes to the protein one by one, one sequence at a time.
MIMOP	2006	PHP	Independent	Upon request	MIMOP provides an environment for mimotope characterization which integrates two main approaches, MimAlign and MimCons, which deliver to the user mimotope analysis results.
MIMOX	2006	Perl	Independent	Web	MIMOX has two sections, the first is to derive the consensus sequence, and the second is to map the single sequence to the target protein.
Mapitope	2007	C++	Windows	Web	Mapitope is based on that epitope determinants shared by the entire set of peptides are detected. Both web service and source code is available.
PepSurf	2007	C++	Linux	Web	PepSurf is an algorithm for mapping a set of affinity-selected peptides to the solved surface of the antigen. Both web service and source code is available.
Pepitope	2007	C++	Windows	Web	Pepitope is a combination algorithm of PepSurf and Mapitope, the web service is available freely on the Pepitope server.
MEPS	2007	Java	Independent	Web	MEPS provides two services, one is to evaluate the likelihood that a given peptide to mimic exposed regions of the protein, and the other one is to generate all peptides of a given length to mimic exposed regions of the protein.
Pep-3D-Search	2008	VB	Windows	Graphic interface	Pep-3D-Search is an epitope mapping algorithm based on both mimotope and motif analysis. The source code is available freely.
EpiSearch	2009	not stated	not stated	Web	EpiSearch is an automated detection of conformational epitopes using random peptide library screening. It provides web service freely.

#### 2.2.1. Criteria and datasets used in methods evaluation

There is no commonly acceptable standard for evaluating mimotope based B-cell epitope prediction methods. Some authors measure only the number of predicted epitope residues and the true epitope residues on a few test cases [40,45]. Some authors measure the sensitivity, positive predictive value (PPV) and Matthews correlation coefficient (MCC) [42]. When approaching this task of evaluating and comparing different prediction methods, a number of problems occur. Using the benchmark dataset to evaluate the methods, we found that some data have several predicted clusters while some have only one cluster, but the highest score cluster does not always perform best. Addressing this problem we defined the best resulting cluster to give a fair comparison for the test sets with different number of resulting clusters. Different methods adopt different algorithms and have different restrictions. Pep-3D-Search is based on the establishment of empirical background distribution for aligning score of every mimotope and antigen, if the P-value of aligning score for every mimotope is bigger than 10^–3^, Pep-3D-Search will not give any prediction result; the online Pepsurf and Pepitope have the restriction that the length of mimotope sequence cannot be longer than 14; and EpiSearch has the restriction that the number of mimotope sequences cannot be longer than 30. Considering these restrictions, we defined a representative dataset for further evaluation of the prediction methods. Being aware of above problems and limitations, we also applied the following performance measures in this study to provide a complete and fair evaluation of the methods:

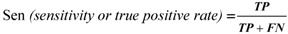
(1)

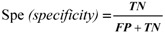
(2)

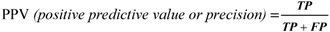
(3)

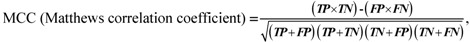
(4)

In our study, TP is the number of predicted epitope residues proven to be the true epitope residues. FP is the number of predicted epitope residues proven not to be the true epitope residues. TN is the predicted non-epitope residues proven not to be the true epitope residues. FN is the number of predicted non-epitope residues proven to be the true epitope residues. In these performance measures, MCC is in essence a correlation coefficient between the identified and the true epitope residues; it returns a value between –1 and +1. The value +1 represents a perfect prediction, the value 0 represents an average random prediction and –1 represents an inverse prediction. 

Because the five epitope prediction methods adopt different strategies to define the surface amino acids of the antigen, to be fair we took the amino acid number of the whole antigen as TP+FP+FN+TN instead of the surface amino acids number for calculating the above performance measures. According to analysis of the testing results with the true epitopes, we found that the first resulting cluster did not always performs best, and then we defined the best resulting cluster as the cluster whose TP is the highest through comparison with the true epitopes in this paper in order to achieve the best performance of the methods for evaluation.

The performance measures were applied in evaluating the methods as follows: all methods were tested on the benchmark dataset and the best resulting cluster which correctly predicted the most epitope residues among the first three resulting clusters was retained. MCC, for all data have been calculated to evaluate the methods through the benchmark dataset; then scatter diagrams of sensitivity with respect to 1-specificity were figured to compare the methods with the random prediction. Finally, the average of all performance measures on the two datasets were calculated to give a direct view of the prediction performance of all methods on different datasets.

#### 2.2.2. Evaluation through MCC

We evaluated the methods through the benchmark dataset using the MCC, and the results are shown in Table 2. For the five methods which evaluated in the paper, only EpiSearch can provide more than three prediction clusters, and the number is unequal, while the other methods all provide three clusters at most. To be fair for evaluation, we retained only the first three highest score clusters for EpiSearch. The all resulting test clusters are provided in Supplementary File 2.

**Figure 1:**
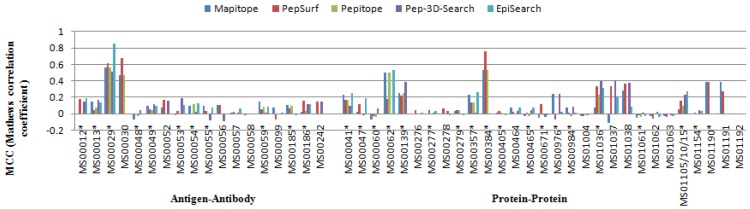
The MCC of each method on the benchmark dataset. The data which belong to the representative dataset were marked with *.

Figure 1 gives the MCC of all methods on the benchmark dataset. Through comparison and analysis we found the following facts: first, small antigens result in several high points. The antigen of MS00012, MS00029, MS00030, MS00062, MS00242, MS00384, MS01190, MS01191 and MS01192 all have less than 30 amino acids. It is not difficult to imagine that under the same conditions a short antigen may give better performance, but in fact so short a antigen is less significant indeed. Secondly, large antigens (more than 500 amino acids) result in low MCC in most of methods, and some values are negative (seen from the low points in the figure). MS00048, MS00099, MS00276, MS00277, MS00278 and MS00279 all belong to this situation, but there is an exception dataset: MS00049, as the performance of every method with it is better. The corresponding target-template complex of MS00049 and MS00099 are the same [PDB id: 1N8Z], then the big difference of MCC may result from the number of mimotope sequence: the MS00049 has five mimotopes and the MS00099 has two. The large number of mimotopes may contain more features of epitope, so the prediction result of MS00049 is relatively good. Lastly, medium antigens (where the number of amino acids ranges from 30 to 500) with the corresponding mimotope sequence number from 1 to 41 result in diversiform MCC values. There are 31 such data in the benchmark datasets. Among these data, MS00058 and MS00060 got low MCC for all methods, but MS00056 and MS00139 obtained high MCC for all methods. Since the length of template chain in these four data is medium, and the number of mimotope sequence is all large, to find the particular reason we did the following analysis.

**Table 2:** MCC and PPV of each method on the benchmark datasets. The data which belong to the representative dataset were marked with *. **(1)** ‘NA’ means that the results for epitopes were not obtained; **(2)** ‘—’ means that the data has restriction of the sequence length or the sequence number; **(3)** The best resulting cluster that was found in the second-ranked cluster is shown in bold; **(4)** The best resulting cluster that was found in the third-ranked cluster is shown in italic; **(5)** “ALL” means the residue number of the antigen.

MimoID	Antigen	ALL	Mapitope				PepSurf		Pepitope		Pep-3D-Search		EpiSearch	
			MCC		PPV		MCC	PPV	MCC	PPV	MCC	PPV	MCC	PPV
**Antigen-Antibody**														
MS00012*	2OSL_P	25	NA	NA		0.182		0.250	NA	NA	0.145	0.200	***0.190***	***0.2*** ***67***
MS00013*	3IU3_I	223	**0.146**	**0.667**		0.045		0.231	**0.071**	**0.500**	**0.168**	**0.471**	***0.134***	***0.*** ***500***
MS00029*	1TET_P	15	0.564	1.000		0.617		1.000	0.564	1.000	0.510	0.900	0.856	1.000
MS00030	1TET_P	15	0.471	1.000		0.679		1.000	0.471	1.000	NA	NA	NA	NA
MS00048*	1YY9_A	624	-0.066	0.000		**-0.003**		**0.000**	-0.033	0.000	***0.049***	***0.400***	-0.005	0.000
MS00049*	1N8Z_C	607	0.100	0.692		**0.059**		**0.455**	**0.041**	**1.000**	**0.114**	**0.500**	***0.096***	***0.48***
MS00052	2ADF_A	196	0.074	0.429		0.164		0.556	——	——	0.157	0.359	NA	NA
MS00053*	2ADF_A	196	-0.015	0.000		0.032		0.167	-0.010	0.000	**0.189**	**0.889**	***0.106***	***0.*** ***5*** ***00***
MS00054*	1IQD_C	156	0.093	0.142		-0.006		0.091	0.113	1.000	0.023	0.130	0.129	0.360
MS00055*	2GHW_A	203	0.100	0.400		0.029		0.208	——	——	-0.082	0.000	**0.080**	**0.320**
MS00056	2GHW_A	203	0.110	0.444		0.110		0.385	——	——	-0.089	0.000	——	——
MS00057	2NY7_G	317	**0.004**	**0.100**		**0.026**		**0.222**	——	——	0.006	0.097	0.062	0.300
MS00058	2NY7_G	317	**-0.015**	**0.000**		——		——	——	——	0.000	0.083	——	——
MS00059*	2NY7_G	317	0.152	0.560		0.052		0.205	0.088	0.556	0.001	0.086	0.085	0.333
MS00099	1N8Z_C	607	**0.076**	**0.600**		-0.066		0.000	NA	NA	**-0.005**	**0.000**	**0.005**	**0.059**
MS00185*	1G9M_G	321	0.102	0.324		0.063		0.226	0.091	0.412	-0.001	0.044	**-0.015**	**0.000**
MS00186*	1E6J_P	210	0.021	0.167		0.158		0.478	0.036	0.333	0.119	0.275	**0.114**	**0.364**
MS00242	2OSL_P	25	NA	NA		0.145		0.200	NA	NA	0.145	0.200	NA	NA
**Protein-Protein**														
MS00041*	1OC0_B	51	0.226	0.364		0.166		0.375	0.166	0.375	0.101	0.310	0.254	0.440
MS00047*	1HX1_B	114	0.028	0.238		0.114		0.360	——	——	-0.022	0.167	0.190	0.480
MS00060*	1WLP_B	138	-0.073	0.000		-0.033		0.160	-0.040	0.000	0.065	0.279	——	——
MS00062*	1WLP_A	25	0.496	0.789		**0.180**		**0.714**	**0.499**	**1.000**	NA	NA	0.530	1.000
MS00139*	1K4U_S	62	0.247	0.778		**0.217**		**1.000**	0.247	0.778	0.391	0.611	——	——
MS00276	2GRX_A	725	***-0.007***	***0.000***		0.041		0.261	-0.006	0.000	**-0.006**	**0.026**	0.004	0.069
MS00277*	2GRX_A	725	-0.007	0.000		**0.039**		**0.429**	-0.006	0.000	0.006	0.071	***0.029***	***0.*** ***222***
MS00278	2GSK_A	590	**-0.008**	**0.000**		**0.070**		**0.545**	-0.003	0.000	**0.038**	**0.263**	-0.018	0.000
MS00279*	2GSK_A	590	**0.033**	**0.184**		**0.047**		**0.400**	**0.047**	**0.400**	**-0.011**	**0.000**	**-0.016**	**0.000**
MS00357*	1FLT_X	95	**0.228**	**0.727**		**0.140**		**0.750**	**0.140**	**0.750**	0.005	0.227	**0.259**	**0.688**
MS00384*	3DOW_B	12	0.527	1.000		0.764		1.000	0.527	1.000	NA	NA	NA	NA
MS00405*	1SHY_A	234	**0.008**	**0.125**		**0.036**		**0.200**	**0.017**	**0.200**	-0.005	0.088	**-0.020**	**0.045**
MS00464	1SQ0_A	214	0.077	0.444		0.021		0.188	NA	NA	0.037	0.194	**0.080**	**0.333**
MS00465*	1SQ0_A	214	-0.029	0.000		0.002		0.133	-0.025	0.000	0.046	0.231	***0.071***	***0.*** ***357***
MS00671*	1D4V_B	163	-0.046	0.000		0.117		0.381	-0.021	0.000	**-0.039**	**0.000**	-0.017	0.083
MS00976*	3BT1_A	135	**0.240**	**0.786**		-0.073		0.114	——	——	0.240	0.593	**0.025**	**0.240**
MS00984*	1EER_A	166	0.078	0.455		0.006		0.250	-0.033	0.000	**0.089**	**0.500**	0.001	0.231
MS01004	1MQ8_B	177	-0.031	0.000		-0.030		0.000	NA	NA	-0.014	0.069	NA	NA
MS01036*	3EZE_B	85	0.071	0.400		0.331		0.917	0.230	0.857	0.393	0.850	***0.313***	***0.*** ***909***
MS01037	3EZE_B	85	-0.109	0.000		0.336		0.727	NA	NA	0.408	0.750	**0.198**	**0.550**
MS01038	3EZE_B	85	0.288	0.543		0.364		0.704	——	——	0.375	0.731	0.086	0.429
MS01061*	1MQ8_B	177	-0.051	0.000		**-0.020**		**0.000**	-0.041	0.000	0.009	0.081	**-0.030**	**0.000**
MS01062	1MQ8_B	177	-0.030	0.000		-0.062		0.000	——	——	0.024	0.135	**-0.041**	**0.000**
MS01063	1MQ8_B	177	**-0.027**	**0.000**		-0.039		0.000	——	——	-0.018	0.065	-0.031	0.000
MS01105/10/15*	1II4_A	155	0.059	0.385		0.163		0.545	0.095	0.571	0.233	0.523	0.274	0.750
MS01154*	1HX1_A	400	-0.006	0.000		***0.010***		***0.111***	-0.004	0.000	0.040	0.156	***0.029***	***0.*** ***154***
MS01190*	1G1S_D	28	0.386	0.750		0.388		0.636	——	——	NA	NA	NA	NA
MS01191	1G1S_D	28	0.386	0.750		0.274		0.556	——	——	NA	NA	NA	NA
MS01192	1G1S_D	28	NA	NA		NA		NA	——	——	NA	NA	NA	NA

We reexamined the data with the number of antigen amino acids ranging from 30 to 500, and the number of mimotope sequence is equal or larger than 3 and less than 30 in the benchmark dataset. Through evaluation, we found that the MCC of the following data are still lower: MS00465, MS00671, MS01061, MS01162 and MS001154. The MS00465 and MS00464 have the same target-template complex, and MS00464 has two mimotope sequences while MS00465 has three, but the overall performance of MS00464 is slightly better through the results of five methods. The similarities of mimotopes and epitopes may become the reason that causes this difference. MS00671 and MS01154 have 163 and 400 amino acids in antigen, 13 and eight mimotopes respectively, and the low MCC may also result by the similarities of mimotopes and epitopes in this situation. MS01061 and MS01062 share the same target-template complex [PDB id: 1MQ8], and the antigen of the two data is neither small nor large: 177 amino acids, and the numbers of mimotopes in these two data are 13 and eight, respectively. Moreover there are another two data have the same target-template complex as MS01061 and MS01062: MS01004 and MS01063, but these two data were removed at the beginning of this step for they have one mimotope. The MCCs to the four data are all low, nearly negative in all methods. The reasons for the low MCCs of these four data seems to be complicated: the small numbers of mimotope sequences, and the low affinities between mimotopes and antibodies, as well as the complicated structures of the antigens.

Through MCC we evaluate the methods through the benchmark dataset, and try to find the reasons for different MCCs in all methods. From the analysis, we find that the small antigens are less significant, and the large antigens with small number of mimotope sequences work worse in all methods due to the fact that they contain relatively less epitope features. For the medium antigen with not a small number of mimotope sequences, the reason for low MCCs may be the similarities of mimotopes and epitopes and the complicated structures of the antigens. In addition, the mimotope sequences cannot reflect the whole features of genuine epitope, for example, a mimotope does not contain structure features of epitopes. Methods which considering other features of epitopes may work better. To sum up, finding the relationship between mimotope sequence and the genuine epitope will be an open problem for further research. 

#### 2.2.3. Evaluation through sensitivity/1-specificity

**Figure 2:**
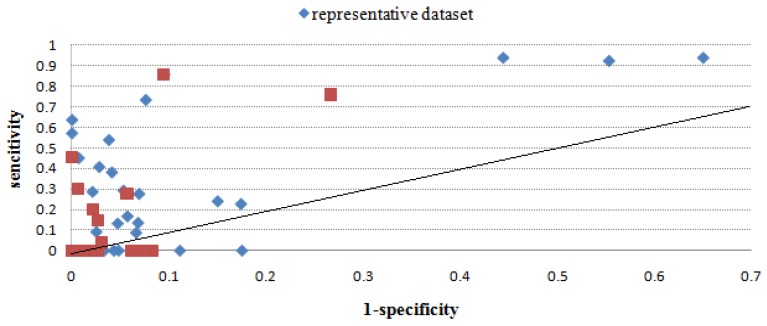
Sensitivity *vs.* 1-specificity scores of Mapitope on the benchmark dataset and the representative dataset.

**Figure 3:**
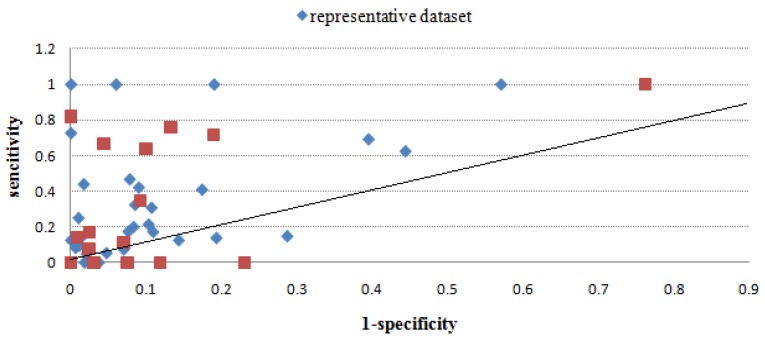
Sensitivity *vs.* 1-specificity scores of PepSurf on the benchmark dataset and the represendative dataset.

**Figure 4:**
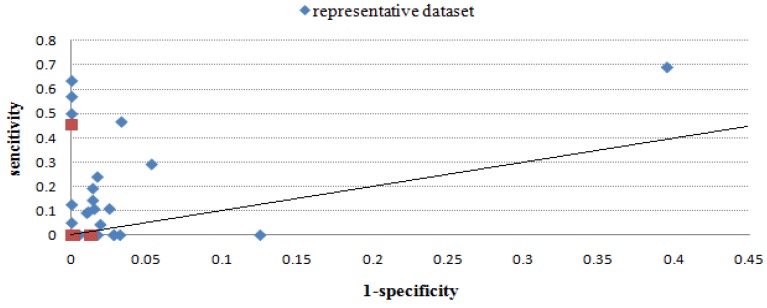
Sensitivity *vs.* 1-specificity scores of Pepitope on the benchmark dataset and the representative dataset.

**Figure 5:**
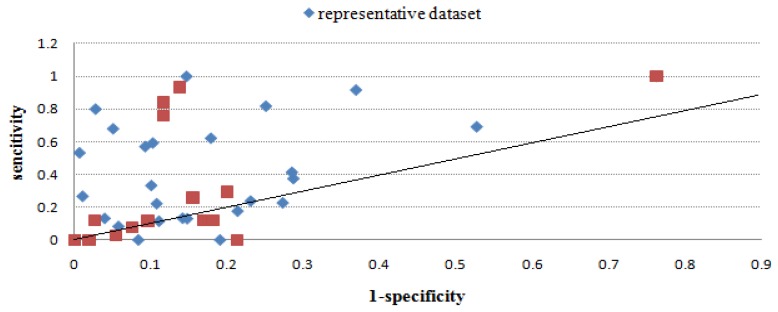
Sensitivity *vs.* 1-specificity scores of Pep-3D-Search on the benchmark dataset and the representative dataset.

**Figure 6:**
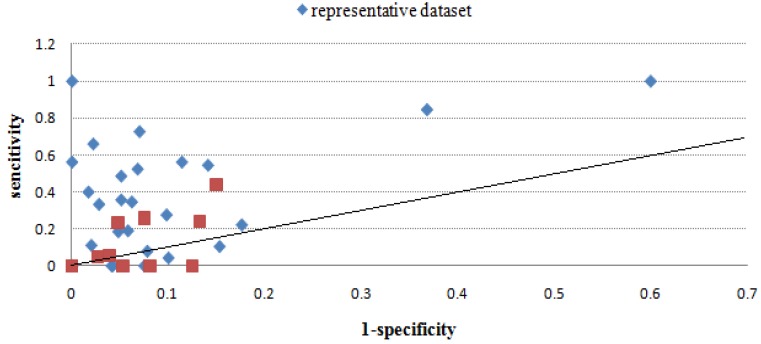
Sensitivity *vs.* 1-specificity scores of EpiSearch on the benchmark dataset and the representative dataset.

Table 3 gives the sensitivity and specificity values of all methods using the benchmark dataset, and Figure 2, Figure 3, Figure 4, Figure 5, Figure 6 give directly relations between sensitivity and 1-specificity of the five methods. For the data whose prediction result is NA or has restrictions to predict, the sensitivity/1-specificity is set to 0. For all methods, there are very few such data that have no prediction result in the representative dataset.

As seen from Figure 2, Figure 3, Figure 4, Figure 5, Figure 6, Mapitope has three points in the origin, 28 points above the diagonal and 16 points below. PepSurf has two points in the origin, 36 points above the diagonal and nine points below. Pepitope is the combined method of Mapitope and PepSurf. Pepitope was tested on the web, and much data gave null results or length restriction of mimotopes. The number of points in the origin is 19 and the number of points above the diagonal is 17 and below is 11. Pep-3D-Search has six points in the origin, 31 points above the diagonal and 10 points below. EpiSearch has the number restriction of mimotope sequences, and then there are 12 points in origin, 25 points above the diagonal and 10 points below. 

**Table 3:** Sensitivity and specificity of each method on the benchmark dataset. The data which belong to the representative dataset were marked with *. **(1)** ‘NA’ means that the results for epitope prediction were not obtained; **(2) **‘—’ means that the data has restriction of the sequence length or the sequence number; **(3)** The best resulting cluster that was found in the second-ranked cluster is shown in bold; **(4)** The best resulting cluster that was found in the third-ranked cluster is shown in italic; **(5)** “EPI” means the residue number of the epitope.

MimoID	Antigen	EPI	Mapitope		PepSurf		Pepitope		Pep-3D-Search		EpiSearch	
			Sen	Spe	Sen	Spe	Sen	Spe	Sen	Spe	Sen	Spe
**Antigen-Antibody**												
MS00012*	2OSL_P	4	NA	NA	1.000	0.429	NA	NA	1.000	0.238	***1.000***	***0.400***
MS00013*	3IU3_I	28	**0.286**	**0.979**	0.214	0.897	**0.107**	**0.985**	**0.571**	**0.908**	***0.357***	***0.949***
MS00029*	1TET_P	11	0.636	1.000	0.727	1.000	0.636	1.000	0.818	0.750	1.000	1.000
MS00030	1TET_P	11	0.455	1.000	0.818	1.000	0.455	1.000	NA	NA	NA	NA
MS00048*	1YY9_A	15	0.000	0.952	**0.000**	**0.982**	0.000	0.984	***0.267***	***0.990***	0.000	0.959
MS00049*	1N8Z_C	20	0.450	0.993	**0.250**	**0.990**	**0.050**	**1.000**	**0.800**	**0.973**	***0.660***	***0.978***
MS00052	2ADF_A	15	0.200	0.978	0.667	0.956	——	——	0.933	0.862	NA	NA
MS00053*	2ADF_A	15	0.000	0.967	0.200	0.917	0.000	0.983	**0.533**	**0.994**	***0.333***	***0.972***
MS00054*	1IQD_C	16	0.938	0.350	0.125	0.857	0.125	1.000	0.375	0.714	0.562	0.886
MS00055*	2GHW_A	29	0.276	0.931	0.172	0.891	——	——	0.000	0.810	**0.276**	**0.902**
MS00056	2GHW_A	29	0.276	0.943	0.345	0.908	——	——	0.000	0.787	——	——
MS00057	2NY7_G	26	**0.038**	**0.969**	**0.077**	**0.976**	——	——	0.115	0.904	0.231	0.952
MS00058	2NY7_G	26	**0.000**	**0.973**	——	——	——	——	0.077	0.924	——	——
MS00059*	2NY7_G	26	0.538	0.962	0.308	0.893	0.192	0.986	0.115	0.890	0.346	0.938
MS00099	1N8Z_C	20	**0.300**	**0.993**	0.000	0.969	NA	NA	**0.000**	**0.981**	**0.050**	**0.973**
MS00185*	1G9M_G	15	0.733	0.924	0.467	0.922	0.467	0.967	0.133	0.859	**0.000**	**0.922**
MS00186*	1E6J_P	11	0.091	0.975	1.000	0.940	0.091	0.990	1.000	0.854	**0.727**	**0.930**
MS00242	2OSL_P	4	NA	NA	1.000	0.238	NA	NA	1.000	0.238	NA	NA
**Protein-Protein**												
MS00041*	1OC0_B	13	0.923	0.447	0.692	0.605	0.692	0.605	0.692	0.474	0.846	0.632
MS00047*	1HX1_B	22	0.227	0.826	0.409	0.826	——	——	0.227	0.728	0.545	0.859
MS00060*	1WLP_B	29	0.000	0.917	0.138	0.807	0.000	0.972	0.414	0.716	——	——
MS00062*	1WLP_A	16	0.938	0.556	**0.625**	**0.556**	**0.500**	**1.000**	NA	NA	0.562	1.000
MS00139*	1K4U_S	24	0.292	0.947	**0.125**	**1.000**	0.292	0.947	0.917	0.632	——	——
MS00276	2GRX_A	36	***0.000***	***0.980***	0.167	0.975	0.000	0.987	**0.028**	**0.946**	0.056	0.961
MS00277*	2GRX_A	36	0.000	0.981	**0.083**	**0.994**	0.000	0.987	0.083	0.943	***0.111***	***0.980***
MS00278	2GSK_A	42	**0.000**	**0.989**	**0.143**	**0.991**	0.000	0.998	**0.119**	**0.974**	0.000	0.947
MS00279*	2GSK_A	42	**0.167**	**0.943**	**0.095**	**0.989**	**0.095**	**0.989**	**0.000**	**0.980**	**0.000**	**0.958**
MS00357*	1FLT_X	21	**0.381**	**0.959**	**0.143**	**0.986**	**0.143**	**0.986**	0.238	0.770	**0.524**	**0.932**
MS00384*	3DOW_B	7	0.571	1.000	1.000	1.000	0.571	1.000	NA	NA	NA	NA
MS00405*	1SHY_A	27	**0.087**	**0.934**	**0.174**	**0.924**	**0.043**	**0.981**	0.130	0.853	**0.043**	**0.900**
MS00464	1SQ0_A	27	0.148	0.973	0.111	0.930	NA	NA	0.259	0.845	**0.259**	**0.925**
MS00465*	1SQ0_A	27	0.000	0.957	0.074	0.930	0.000	0.968	0.222	0.893	***0.185***	***0.952***
MS00671*	1D4V_B	19	0.000	0.889	0.421	0.910	0.000	0.972	**0.000**	**0.917**	0.105	0.847
MS00976*	3BT1_A	27	**0.407**	**0.972**	0.148	0.713	——	——	0.593	0.898	**0.222**	**0.824**
MS00984*	1EER_A	38	0.132	0.953	0.053	0.953	0.000	0.984	**0.132**	**0.961**	0.079	0.922
MS01004	1MQ8_B	17	0.000	0.919	0.000	0.925	NA	NA	0.118	0.831	NA	NA
MS01036*	3EZE_B	25	0.240	0.850	0.440	0.983	0.240	0.983	0.680	0.950	***0.400***	***0.983***
MS01037	3EZE_B	25	0.000	0.917	0.640	0.900	NA	NA	0.840	0.883	**0.440**	**0.850**
MS01038	3EZE_B	25	0.760	0.733	0.760	0.867	——	——	0.760	0.883	0.240	0.867
MS01061*	1MQ8_B	17	0.000	0.825	**0.000**	**0.963**	0.000	0.875	0.176	0.787	**0.000**	**0.925**
MS01062	1MQ8_B	17	0.000	0.925	0.000	0.769	——	——	0.294	0.800	**0.000**	**0.875**
MS01063	1MQ8_B	17	**0.000**	**0.938**	0.000	0.881	——	——	0.118	0.819	0.000	0.919
MS01105/10/15*	1II4_A	37	0.135	0.932	0.324	0.915	0.108	0.975	0.622	0.822	0.486	0.949
MS01154*	1HX1_A	21	0.000	0.989	***0.048***	***0.979***	0.000	0.995	0.333	0.900	***0.190***	***0.942***
MS01190*	1G1S_D	7	0.857	0.905	1.000	0.810	——	——	NA	NA	NA	NA
MS01191	1G1S_D	7	0.857	0.905	0.714	0.810	——	——	NA	NA	NA	NA
MS01192	1G1S_D	7	NA	NA	NA	NA	——	——	NA	NA	NA	NA

Through the scatter diagrams of sensitivity with respect to 1-specificity, we evaluated the methods with the random prediction. From the analysis above we can see that for most test cases the five epitope prediction methods can all precisely localize epitope regions. The predicted results of all methods are totally better than random prediction. But due to the restrictions of each methods and the mimotope sequence itself, there is still distance from a satisfactory predicting precision. Moreover, to make a systematic evaluation of epitope prediction methods, the quantity and diversity of this benchmark dataset is still not enough, more reliable experiment data are expected.

#### 2.2.4. Overall performance evaluation

We calculated the average value of sensitivity, specificity, PPV and MCC using the benchmark dataset and the representative dataset. Meanwhile, we also gave the average values of these performance measures using antigen-antibody and protein-protein interactions for each method respectively. Table 4 gives the overall performance for each method.

**Table 4:** Overall performance of each method.

Statistics	Mapitope	PepSurf	Pepitope	Pep-3D-Search	EpiSearch
Antigen-Antibody					
sensitivity	0.326	0.434	0.212	0.455	0.426
specificity	0.931	0.869	0.990	0.804	0.905
PPV	0.407	0.334	0.580	0.273	0.345
MCC	0.120	0.134	0.143	0.085	0.141
Protein-Protein					
sensitivity	0.254	0.305	0.149	0.333	0.241
specificity	0.895	0.889	0.956	0.842	0.907
PPV	0.311	0.409	0.330	0.288	0.317
MCC	0.105	0.127	0.099	0.099	0.099
Benchmark dataset (Representative dataset)					
sensitivity	0.280(0.320)	0.339(0.326)	0.172(0.174)	0.368(0.387)	0.289(0.342)
specificity	0.908(0.890)	0.892(0.901)	0.968(0.965)	0.841(0.845)	0.921(0.922)
PPV	0.346(0.377)	0.384(0.398)	0.419(0.429)	0.284(0.322)	0.329(0.378)
MCC	0.112(0.127)	0.129(0.126)	0.116(0.112)	0.092(0.101)	0.112(0.139)

**Figure 7:**
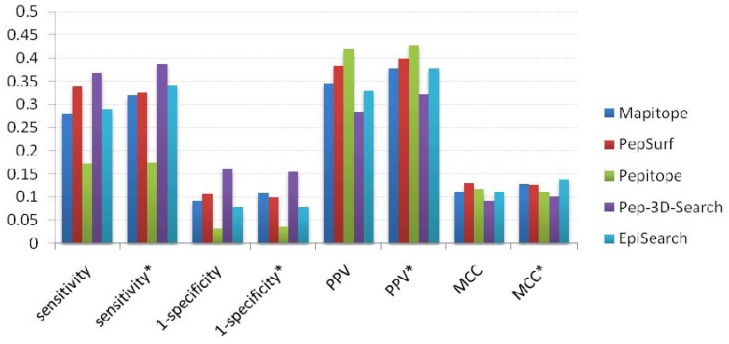
Overall performancesofeach method. The average values of sensitivity, specificity, PPV and MCC were calculated for each method using the benchmark dataset and the representative dataset. The performance measures of the representative dataset are marked with *.

As one can see in Table 4, when using the benchmark dataset, Pep-3D-Search gives the best sensitivity but low PPV and MCC; this resulted from the number of the positively predicted epitope residues being large as well as the total number of predicted epitope residues being even larger so more false positive residues are included. Pepitope gives the best specificity and PPV because it is a combination algorithm of Mapitope and PepSurf, and the total number of predicted epitope residues is small. The performance measures of EpiSearch fall in the middle of the five methods. As seen from Figure 8, PepSurf was rated best with a MCC value of 0.129. The other four methods performed with a MCC about 0.1. The best performances of these methods were Pep-3D-Search with a sensitivity of 0.368 and PPV of 0.284 and PepSurf with a sensitivity of 0.339 and PPV of 0.384. Using the representative dataset, the sensitivity and PPV of each method are slightly improved, but the specificity and MCC are more or less the same with using the benchmark dataset (see Figure 7).

Seen from this result, the overall performance was poor for all methods. When testing the methods using the representative dataset, the performances of all methods are just slightly improved. The poor performances results from two aspects. The one is the method itself. These five methods have different restrictions. The restrictions lead to the methods not working with some test cases. The other aspect for the poor performance is the dataset itself. Seen from the performance measures of every data in the benchmark dataset (Table 2 and Table 3), there are some data that gave satisfactory results in all methods, whereas the opposite is true for others. This is because the mimotope has low similarity with the antigen, so it contains few features of the genuine epitopes, thus using the mimotope to map epitopes will not give a satisfactory result. To improve the performance of epitope prediction based on random peptide library screening, the improvement in both mimotopes and algorithms are required.

## 3. Materials and Methods

### 3.1. Construction of the Datasets

The datasets were derived from MimoDB and PDB:

Benchmark dataset—test sets with corresponding 3D structure of template-target complex. This dataset is intended for evaluation the performance of existing epitope prediction methods to each other and development of new method. The benchmark dataset contains 47 test sets with 28 3D structures of template-target complex. 

Representative dataset—a subset of the benchmark dataset. It contains only one test set for each template-target complex in the benchmark dataset. This dataset is intended for further verifying the validity of the epitope prediction methods and accessing the performance of the methods. The representative dataset contains 30 test sets with 28 3D structures of template-target complex. The following steps 1-4 relate to the benchmark dataset; steps 1–5 relate to the representative dataset. 

*Step 1*: 62 test sets which are related to 53 solved structures of template-target complex were collected from MimoDB. The corresponding crystal structure of template-target complexes were downloaded from the PDB. If a test set has several complex structures we selected one according to the resolution and release date. Among these test sets, there are 18 sets with solved 3D structures of Ag-Ab complex, and 44 sets with solved 3D structures of protein-protein complex. The five methods evaluated in the paper all developed for epitope prediction, and the test sets with the protein-protein interactions could be taken as supplementary data to verify the efficiency of the methods. The number of peptide sequence in these test sets is range from 1 to 41. 

*Step 2*: all the test sets were checked and filtered carefully in this step. The test sets with either different library names or different experimental methods were considered as different sets. The test sets which were published in different papers by different research teams were considered as different, while the ones which published in different papers by the same research team were combined as one test set. For the test sets with different round of biopanning in experiment and the other conditions are the same, we choose the last round one. The MS01153 and MS01154 ones were in this situation. The MS01153 was obtained from two rounds of biopanning while the MS01154 was obtained after three rounds of biopanning, so the MS01153 was excluded. The test sets from MS01101 to MS01115 were slightly more complicated. The template of these sets was the same and the targets were obtained in three different ways, and these 15 data sets were obtained according five rounds of biopanning. Through background knowledge we choose the last round ones as results, therefore the MS01105, MS01110 and MS01115 were retained and the others were excluded. The target of these three sets were the same antibody from different organisms rather than different antibodies, thus we combined these three data sets as one test set-MS01105/10/15. Then we checked the sequence identity of the test sets whose number of peptide sequence is more than 20. There are six such test sets. Through pairwise alignment for each set and taking the identity of 70% as the threshold, we found that there are no more than three identity sequences. Then we excluded one sequence from each identity peptide sequence pairs according to the peptide binding affinity, and the mimotope sequences in following three sets changed: MS00054, MS00058 and MS01105/10/15. After this step, 47 test sets were retained.

*Step 3*: for each 3D structure of template-target complex, the template chain was extracted and the target chain which interacts with the template was recorded to locate the epitopes. There are two cases: the first is the 3D structure has one template chain, and we extracted the single chain directly and recorded the target chain in this situation; the second situation is the 3D structure has several template-target pairs, since the template and target are identical and the binding sites are the same, so we choose one pair randomly and extracted the template chain and recorded the target chain.

*Step 4*: for each test set, the mimotope sequences, the 3D structure of template-target complex and the structure of template are all ready. In this step the epitopes were determined. We confirmed the epitopes through three tools: CED [53], IEDB-3D [54] and CMA (Contact Map Analysis) [55]. The definitions of an epitope inferred from the 3D structure of Ag-Ab complex are mainly based on either ASA (accessible surface area) or the contact area between residues of antigen and antibody. In this study, we define an epitope as the residues of an antigen which has a contact area above 4 Å^2^ upon interaction with the antibody. As this definition, when applying CMA to locate the epitopes the contact area threshold is setting 4 Å^2 ^and the other parameters are setting as default. We adopted the same way to locate the binding-sites of protein-protein interactions. In this way, 47 data sets with corresponding mimotope sequences, 28 3D structures of template-target complexes, structures of template chains and the genuine epitope residues are all determined. The resulting dataset is denoted as the benchmark dataset. The benchmark dataset is listed in Table 5. The detailed datasets are given in the Supplementary Material section.

**Table 5:** The dataset compiled from MimoDB, the data which belong to the representative dataset were marked with *. **(1)** Number of peptides ×peptide length. **(2)** PMID of the reference. **(3)** The combination of MS01105, MS01110 and MS01115.

Mimo_ID	PDB_ID	Template	Target	Library(1)	Ref (2)
**Antigen-Antibody**					
MS00012*	2OSL	B-lymphocyte antigen CD20	Anti-CD20 monoclonal antibody rituximab	13 × 9	16705086
MS00013*	3IU3	Interleukin-2 receptor subunit alpha	Anti-CD25 monoclonal antibody basiliximab	6 × 9	17440057
MS00029*	1TET	Heat-labile enterotoxin B chain	Anti-LTP-B monoclonal antibody TE33	10 × 9	16273596
MS00030	1TET	Heat-labile enterotoxin B chain	Anti-LTP-B monoclonal antibody TE33	5 × 11	16273596
MS00048*	1YY9	Epidermal growth factor receptor	Cetuximab	4 × 12	16288119
MS00049*	1N8Z	Receptor tyrosine-protein kinase erbB-2	Trastuzumab	5 × 12	15210798
MS00052	2ADF	von Willebrand factor	Anti-vWF monoclonal antibody 82D6A3	2 × 15	12855771
MS00053*	2ADF	von Willebrand factor	Anti-vWF monoclonal antibody 82D6A3	3 × 8	12855771
MS00054*	1IQD	Coagulation factor VIII	Anti-coagulation factor VIII monoclonal antibody BO2C11	27 × 12	12676786
MS00055*	2GHW	Spike glycoprotein	Anti-spike glycoprotein monoclonal antibody 80R	18 × 15	16630634
MS00056	2GHW	Spike glycoprotein	Anti-spike glycoprotein monoclonal antibody 80R	9 × 16, 9 × 15, 19 × 14, 4 × 13	16630634
MS00057	2NY7	Surface protein gp120	Anti-gp120 monoclonal antibody b12	1 × 12, 1 × 15	16940148
MS00058	2NY7	Surface protein gp120	Anti-gp120 monoclonal antibody b12	1 × 6, 1 × 12, 1 × 13, 1 × 16, 1 × 18, 2 × 14, 2 × 20, 2 × 22 8 × 15, 13 × 21	16940148
MS00059*	2NY7	Surface protein gp120	Anti-gp120 monoclonal antibody b12	1 × 10, 1 × 13, 17 × 14	16940148
MS00099	1N8Z	Receptor tyrosine-protein kinase erbB-2	Trastuzumab	2 × 12	15536075
MS00185*	1G9M	Envelope glycoprotein gp120	Anti-gp120 monoclonal antibody 17b	10 × 12, 1 × 10	14596802
MS00186*	1E6J	Capsid protein p24	Anti-p42 monoclonal antibody 13b5	14 × 14, 2 × 7	14596802
MS00242	2OSL	B-lymphocyte antigen CD20	Anti-CD20 monoclonal antibody rituximab	7 × 12	16814270
**Protein-Protein **					
MS00041*	1OC0	Vitronectin	Plasminogen activator inhibitor 1	8 × 13, 1 × 7, 1 × 11	16813566
MS00047*	1HX1	BAG family molecular chaperone regulator 1	Heat shock cognate 71 kDa protein	8 × 15	7649995
MS00060*	1WLP	Neutrophil cytosol factor 1	Cytochrome b-245	2 × 8, 31 × 9	7592831
MS00062*	1WLP	Cytochrome b-245 light chain	Neutrophil cytosol factor 1	4 × 5, 3 × 9, 1 × 10, 1 × 8	7624379
MS00139*	1K4U	Neutrophil cytosol factor 1	Neutrophil cytosol factor 2	28 × 9, 2 × 10, 4 × 12, 2 × 6, 1 × 8	8663333
MS00276	2GRX	Ferrichrome-iron receptor	Protein tonB	12 × 12	16414071
MS00277*	2GRX	Ferrichrome-iron receptor	Protein tonB	6 × 9	16414071
MS00278	2GSK	Vitamin B12 transporter btuB	Protein tonB	2 × 12	16414071
MS00279*	2GSK	Vitamin B12 transporter btuB	Protein tonB	6 × 9	16414071
MS00357*	1FLT	Vascular endothelial growth factor receptor 1	Vascular endothelial growth factor A	4 × 7	17401149
MS00384*	3DOW	Calreticulin	Gamma-aminobutyric acid receptor-associated protein	5 × 12	17916189
MS00405*	1SHY	Hepatocyte growth factor	Hepatocyte growth factor receptor	2 × 12, 1 × 13	17947467
MS00464	1SQ0	von Willebrand factor	Platelet glycoprotein Ib alpha chain	2 × 11	18363340
MS00465*	1SQ0	von Willebrand factor	Platelet glycoprotein Ib alpha chain	3 ×11	18363340
MS00671*	1D4V	Tumor necrosis factor ligand superfamily member 10	Tumor necrosis factor receptor superfamily member 10B	13 × 9	20156289
MS00976*	3BT1	Urokinase-type plasminogen activator	Urokinase plasminogen activator surface receptor	19 × 15	8041758
MS00984*	1EER	Erythropoietin	Erythropoietin receptor	1 × 10	8662529
MS01004	1MQ8	Integrin alpha-L beta-2	Intercellular adhesion molecule 1	1 × 14	8953648
MS01036*	3EZE	Phosphocarrier protein HPr	Phosphoenolpyruvate-protein phosphotransferase	11 × 6	9350871
MS01037	3EZE	Phosphocarrier protein HPr	Phosphoenolpyruvate-protein phosphotransferase	9 × 10	9350871
MS01038	3EZE	Phosphocarrier protein HPr	Phosphoenolpyruvate-protein phosphotransferase	6 × 15	9350871
MS01061*	1MQ8	Integrin alpha-L beta-2	Intercellular adhesion molecule 1	12 × 9, 1 × 8	11532073
MS01062	1MQ8	Integrin alpha-L beta-2	Intercellular adhesion molecule 1	1 × 9, 7 × 16	12963036
MS01063	1MQ8	Integrin alpha-L beta-2	Intercellular adhesion molecule 1	1 × 16	12963036
MS01105/10/15*(3)	1II4	Heparin-binding growth factor 2	Fibroblast growth factor receptor 2	30 × 7	12032665
MS01154*	1HX1	Heat shock cognate 71 kDa protein	BAG family molecular chaperone regulator 1	8 × 12	11121403
MS01190*	1G1S	P-selectin glycoprotein ligand 1	P-selectin	5 × 17	12393589
MS01191	1G1S	P-selectin glycoprotein ligand 1	P-selectin	2 × 15	12393589
MS01192	1G1S	P-selectin glycoprotein ligand 1	P-selectin	1 × 13, 1 × 18	12393589

*Step 5*: in the benchmark dataset, there exist some test sets have the same template-target complex, but these test sets cannot be combined because of different experimental methods or different library names used by different research teams. For one complex structure, we retained only one test set which has better prediction results for most methods and excluded the others. The same structures of complex but different template chain are taken as different (there are two such complexes in the 28: 1HX1 and 1WLP). In this way, 30 test sets with 28 3D structures of template-target complexes were collected. The final dataset is defined as the representative dataset.

### 3.2. Algorithm Evaluation

We consulted all epitope mapping algorithms based on random peptide library screening published thus far, to the best of our knowledge (Table 1). All methods were identified through PubMed and web search. Through comparative analysis we chose five of them for evaluation based on our benchmark dataset. All these five tools are either open source or provide friendly web service freely. In addition, we adopted the default parameters provided by each tool in all test sets.

Mapitope is based on an alternative computational approach in which epitope determinants shared by the entire set of peptides are detected. Mapitope defines AAP (pairs of amino acids) by calculating the distance between two α carbon atoms of adjacent residues. Each peptide sequence obtained from the phage library is translated into AAPs, and then Mapitope calculates them to rank the occurrences of AAPs to obtain a set of major SSP (statistically significant pairs), and finally uses them to search the 3D structure of the antigen and links the SSP into clusters on the antigen surface as predicted epitope. Mapitope algorithm is implemented in C++, and we get the source code and binaries freely on line, so this algorithm is tested in local environment. The MS00139 gave no result when tested on a local computer, but gave results when tested online. The result of MS00139 is obtained on web service while the results of other sets are obtained locally. 

PepSurf is an algorithm for mapping a set of affinity-selected peptides onto the solved surface of the antigen. This is done by efficiently searching virtually all possible 3D paths based on the color-coding technique for those that exhibit high similarity to the peptide sequences. A modified BLOSUM62 matrix is used for scoring amino acid similarities in mapping step. Then the best alignment of each peptide to antigen residues brought to proximity by folding is obtained. The resulting most significant alignments are then clustered and the epitope location is inferred. The PepSurf algorithm is also implemented in C++, and the source code and binaries are freely available online. To avoid the length restriction of peptides sequence (shorter than 15 amino acids) online, we tested PepSurf locally. The result of MS1105/10/15 is obtained online since there was no result when tested locally. But the situation is unfortunate for MS00058. MS00058 also has no result when tested locally, and it has 32 mimotope sequences while 27 of them have the sequence length exceed 14, so the web service is also powerless.

Pepitope is a web-based tool server that aims at predicting B-cell epitopes based on random peptide library screening; it provides three different algorithms for epitope mapping: Mapitope, PepSurf and a combination of the two. The predicted clusters in combination algorithm include only residues which were predicted to be part of the epitope in both algorithms. In this paper we referred Pepitope as the combination algorithm. The web service of Pepitope is available freely on the Pepitope server. The Pepitope algorithm is only available online, and the online service also has the length restriction of peptides sequence shorter than 15 amino acids, and 13 sets of the benchmark datasets thus had no results.

Pep-3D-Search is an epitope mapping algorithm based on both mimotope and motif analysis. An ACO (Ant Colony Optimization) algorithm was proposed for aligning a 1D mimotope sequence (or a motif sequence) to the 3D structure of an antigen, and P-value calculation based screening strategy and DFS (Depth-First Search) algorithm based clustering strategy were employed in localizing epitope candidate regions. Pep-3D-Search is implemented in VB and the source code and executable program can be freely obtained. We tested the benchmark dataset established in this paper locally with Pep-3D-Search. Since the algorithm is based on the establishment of empirical background distribution for aligning score of every mimotope and antigen, it cannot work with data whose P-value of the aligning score of every mimotope is more than 10^−3^. The MS00030, MS00062, MS00384, MS01190, MS01191 and MS01192 fall into this group and gave no results. Pep-3D-Search also provides the selection of mapping epitope based on motif analysis. The output can give 10 best fits. When the number of mimotope sequences is less than three the motif would not be gained and there is no result returned. MS00057, MS00099, MS00278 and MS00464 all have two mimotope sequences while MS01004 and MS01063 have one mimotope sequence, so there is no result for these data. Among all algorithms evaluated in this paper, only Pep-3D-Search supporsts the epitope mapping based on motif, so we do not give an evaluation of Pep-3D-Search results which are based on motif analysis with the other algorithms, but the testing results are provided in Supplementary File 2.

EpiSearch is based on a patch analysis that identifies spatial contiguous clusters of residues on the surface of the antigen with similar physicochemical properties as found in the mimotopes. The amino acid compositions of the 1D and 3D profiles are compared through three matrixes and quantified in a score function for each patch on the protein surface. Similarity of residues is measured by a physical-chemical property distance that was derived from five descriptors of amino acid residues. The highest scoring patches are listed in the output files and are also displayed on the surface of the protein. The benchmark dataset is tested with the online version of the EpiSearch method. EpiSearch has a peptide sequence number restriction, so it cannot work with more than 30 mimotope sequences at one time. MS00056, MS00058, MS00060 and MS00139 all have mimotope sequences of more than 30 so they returned no results. 

## 4. Conclusions

B-cell epitope prediction is important for vaccine design, development of diagnostic reagents and for studies to elucidate the interactions between antigen and antibody on a molecular level. Epitope prediction methods based on random peptide library screening are supposed to tremendously improve the accuracy of the epitope prediction by combining experiments with computational methods, and thus have attracted the attention of many researchers. In this paper, a benchmark dataset for evaluating B-cell epitope prediction methods based on random peptide library screening has been constructed and was made available. Using this benchmark dataset and a representative dataset, five publicly available methods were evaluated. Several schemes were implemented for evaluating the methods.

Firstly, we evaluated the methods through the MCC measure using the benchmark dataset, and tried to find what kind of data is more suitable for further development of epitope prediction based on random peptide library screening. We find that the number of antigen amino acids and the number of mimotopes influenced the prediction result, and the relationship between mimotope and the genuine epitope may be another factor which influences the prediction performance. Secondly, the sensitivity with respect to 1-specificity of each method was figured to evaluate the methods and compare them with the random prediction. Through comparison and evaluation, the performances of all methods are superior to random prediction, but the results are still unsatisfactory on both datasets due to diverse restrictions of each method and poor precision. Finally, the average sensitivity, specificity, PPV and MCC of all methods were computed using the two datasets to give a view of the overall performance for all evaluating methods. The overall performance of all methods was poor: using the benchmark dataset they did not exceed 42% precision and 37% sensitivity, and the values of average MCC were about 0.11 for Mapitope, Pepitope and EpiSearch, about 0.09 for Pep-3D-Search, and about 0.13 for PepSurf; while using the representative dataset, the average values of the sensitivity and precision improved a little, while the average values of specificity and MCC are nearly the same. 

Nevertheless, the epitope prediction problem using phage display library screening is far from resolved. Usually a mimotope is a set of peptides sequence, while these sequences cannot reflect the real structure features of genuine epitopes. Though a mimotope has functional similarity with the genuine epitope, and this similarity may be reflected by the physic-chemical properties rather than the sequence similarity. In addition, the available data are still limited for both method evaluation and new algorithm development. Finding the relationship between the mimotopes and the genuine epitopes of antigens is still an open problem for further research. 

Given the current results and the shortcomings of the existing methods, how can epitope prediction be further improved? On the one hand, finding the correlation between mimotope and epitope and obtaining enough mimotopes by designing appropriate experiments is required. On the other hand, the new algorithms for mapping protein epitopes based mimotope are desirable. The interaction sites of an antigen and an antibody are usually a spacial region but not a stretch of sequence, and the epitope prediction based on mimotope analysis is different from the classical sequence aligning. It is possible for improving the prediction performance to combine more features that discriminate epitopes from non-epitopes besides sequence similarity, for example, the evolutionary conservation score, side-chain energy score and planarity score. The application of these features, in the context of conformational epitope prediction, has been raised by others [56]. In addition, for finding the correlation between mimotopes and genuine epitopes based on machine learning methods, it is pre-requisite that larger dataset which is used for training are available. Therefore, more experiment data including available mimotopes and corrsponding structure of template-target complex are strongly needed.

## Additional Materials

### Supplementary File 1

The representative test sets with the sequence of mimotope, the template-target complexes and the represented epitopes. The data provides curate information on 48 test sets with the corresponding 28 3D structure of template-target complexes available in the MimoDB and PDB of November, 2010 and used in this work. Please see the Supplementary Material.

### Supplementary File 2

The detail results on the prediction for the benchmark dataset. This data include the results of all methods. For Pep-3D-Search especially, the data has the prediction results that based on motif analysis. For EpiSearch, first three solutions with the highest score were retained as the results and the results of other methods were all retained. Please see the Supplementary Material.
