# Supplementary material for: Epitope Prediction Based on Random Peptide Library Screening: Benchmark Dataset and Prediction Tools Evaluation
Source: Molecules. 2011 Jun 16;16(6):4971–93. doi: 10.3390/molecules16064971 (PMC6264216; doi:10.3390/molecules16064971)
Supplement: Supplementary file 1 [file molecules-16-04971-s001.zip › supplementary materials/results/pepitope/results.doc]

| **MimoDB ID** | **PDB ID** | **epitope** | **Pepitope** |
| --- | --- | --- | --- |
| MS00012 | 2OSL_P | E168 P169 A170 N171 | NULL |
| MS00013 | 3IU3_I | E1 L2 C3 D4 D5 D6 E22 G23 M25 N27 E29 R36 K38 S39 G40 S41 L42 Y43 L45 T47 G48 N49 S54 D56 N57 I118 Y119 H120 | best: Y129 T14 Y128 V132 C131 Q130  second: Y119 W156 L2 V146 H120 F121 |
| MS00029 | 1TET_P | V3 P4 G5 S6 Q7 H8 I9 D10 S11 Q12 K13 | the best: G5 P4 V3 I9 Q7 S6 H8  second: D10 |
| MS00030 | 1TET_P | V3 P4 G5 S6 Q7 H8 I9 D10 S11 Q12 K13 | G5 V3 P4 S6 Q7 |
| MS00041 | 1OC0_B | C9 T10 E11 G12 F13 D22 E23 L24 C25 Y27 Y28 Q29 S30 | D16 D34 E11 C19 T36 G12 Y35 N14 Q20 Q29 D22 T10 Y28 C31  S30 C39 E38 T33 R8 C9 K17 A37 V15 F13 |
| MS00047 | 1HX1_B | R205 K208 A209 I211 E212 M215 K216 L218 E219 D222 T223 L224 I225 E226 R234 R237 C238 V241 C242 Q245 L248 D252 | Length restriction |
| MS00048 | 1YY9_A | R377Q408 Q432 H433 F436 V441 S442 S464 K467 K489 I491 S492 N497 | Q47 Q8 M30 C7 F20 S26 R29 D22 Q28 L17  second：Y602 T601 C596 Y586 K585 W584 G603 C604 |
| MS00049 | 1N8Z_C | P579 E580 A581 D582 Q583 D592 P593 P594 F595 K615 F616 P617 D618 E619 E620 G621 A622 C623 Q624 P625 | T268 F236 Y267 A271 G270 F269  second: D570 |
| MS00052 | 2ADF_A | I1738 T1739 T1740 I1741 D1742 P1744 W1745 N1746 D1772 F1776 R1779 M1785 H1786 | Length restriction |
| MS00053 | 2ADF_A | I1738 T1739 T1740 I1741 D1742 P1744 W1745 N1746 D1772 F1776 R1779 M1785 H1786 | K1107 C1109 L1105 |
| MS00054 | 1IQD_C | F2215 T2216 N2217 M2218 F2219 R2234 R2239 Q2241 V2242 S2269 L2270 L2271 T2272 H2334 Q2335 | E2228 V2314 Q2266 T2241 N2225 N2224 Q2284 V2223 W2313 P2226  K2227 R2307 S2312 K2258 C2296 R2304 H2309 K2236 N2295  Q2316 W2271 G2268 |
| MS00055 | 2GHW_A | R426 S432 T433 Y436 N437 K439 Y440 Y442 P469 P470 A471 L472 N473 C474 Y475 W476 L478 N479 D480 Y481 G482 Y484 T485 T486 T487 G488 I489 Y491 Q492 | Length restriction |
| MS00056 | 2GHW_A | R426 S432 T433 Y436 N437 K439 Y440 Y442 P469 P470 A471 L472 N473 C474 Y475 W476 L478 N479 D480 Y481 G482 Y484 T485 T486 T487 G488 I489 Y491 Q492 | Length restriction |
| MS00057 | 2NY7_G | S257 N280 A281 K282 S365 G366 G367 D368 P369 E370 I371 V372 T373 Y384 N386 P417 R419 V430 G431 K432 T455 R456 G472 G473 D474 M475 | Length restriction |
| MS00058 | 2NY7_G | S257 N280 A281 K282 S365 G366 G367 D368 P369 E370 I371 V372 T373 Y384 N386 P417 R419 V430 G431 K432 T455 R456 G472 G473 D474 M475 | Length restriction |
| MS00059 | 2NY7_G | S257 N280 A281 K282 S365 G366 G367 D368 P369 E370 I371 V372 T373 Y384 N386 P417 R419 V430 G431 K432 T455 R456 G472 G473 D474 M475 | T384 T373 F277 S257 S274 D474 E370 C275 C418  second: C205 L111 E211 F210 Y435 E106 S110 P206 I424 C109  third: V101 E102 W95 D99 |
| MS00060 | 1WLP_B | I164 A165 Y167 T170 S171 S173 E174 V186 E187 S191 G192 W193 F195 W204 P206 S208 F209 E211 K235 E241 D243 E244 D261 G262 W263 Y274 P276 M278 Y279 | K258 H257 P222 |
| MS00062 | 1WLP_A | S5 K6 Q7 P8 P9 S10 N11 P12 P13 P14 R15 P16 P17 E19 A20 P24 | P24 R15 A20 P17 K23 K22 E19 P14 A18 R21 P16  second: P13 N11 P12 S10 K6 Q7 P9 P8 |
| MS00099 | 1N8Z_C | P579 E580 A581 D582 Q583 D592 P593 P594 F595 K615 F616 P617 D618 E619 E620 G621 A622 C623 Q624 P625 | null |
| MS00139 | 1K4U_S | L465 F466 S467 Y468 Q472 P473 E474 S475 E480 L487 S488 K489 V490 N491 E493 W494 E496 K502 V503 G504 I505 P507 V509 F510 | V509 F510 E492 K489 W494 N491 K508 E493 V490 |
| MS00185 | 1G9M_G | C119 K121 V200 T202 Q203 A204 C205 R419 K421 Q422 I423 K432 M434 Y435 P437 | T123 M434 M426 K121 W427 Q428 I424 N425 L122 G431 K429 K421  Q422 I420 K432 R419 I423  second: P417 |
| MS00186 | 1E6J_P | A204 L205 G206 P207 A208 A209 T210 E212 E213 T216 A217 | D197 C198 A217 |
| MS00242 | 2OSL_P | E168 P169 A170 N171 | NULL |
| MS00276 | 2GRX_A | T8 I9 T10 V11 T12 A13 A14 P15 A16 P17 A26 P27 E56 T158 E159 P160 K162 D186 D542 R543 P544 A588 S590 A591 N594 F634 D635 S639 G640 D676 A678 G681 A683 E684 R724 F725 | S527 T557 S514 N559 Q469 P526 N558 P513 S515 |
| MS00277 | 2GRX_A | T8 I9 T10 V11 T12 A13 A14 P15 A16 P17 A26 P27 E56 T158 E159 P160 K162 D186 D542 R543 P544 A588 S590 A591 N594 F634 D635 S639 G640 D676 A678 G681 A683 E684 R724 F725 | A335 A333 P334 L332 A331 L399 A330 S319 C318 |
| MS00278 | 2GSK_A | P5 D6 T7 L8 V9 V10 T11 A12 N13 R14 E16 P18 R19 V22 L23 Q32 E259 L260 K262 I305 G307 H308 G309 L345 Q347 T352 E378 E381 G382 R384 I386 E423 G424 L425 G428 N430 N469 F470 D471 G473 P474 T476 | P238 |
| MS00279 | 2GSK_A | P5 D6 T7 L8 V9 V10 T11 A12 N13 R14 E16 P18 R19 V22 L23 Q32 E259 L260 K262 I305 G307 H308 G309 L345 Q347 T352 E378 E381 G382 R384 I386 E423 G424 L425 G428 N430 N469 F470 D471 G473 P474 T476 | S237 A190 S287 P238 T188 Q191 P235 Q189  second: A24 S20 F15 P25 Q17 R19 T21 E16 P18 V22  third: Q158 Q157 G137 P136 Q159 |
| MS00357 | 1FLT_X | Y139 E141 I142 P143 I145 H147 K171 F172 P173 Y199 K200 I202 G203 L204 N219 L221 T222 H223 R224 Q225 T226 | K190 R189 S188 P157  second: L174 K171 F172 P173 |
| MS00384 | 3DOW_B | D5 W6 D7 F8 L9 P10 P11 | P11 P10 F8 L9 |
| MS00405 | 1SHY_A | Y513 K516 R533 Q534 P537 R539 D578 Y619 R647 K649 E656 C669 E670 Y673 V692 P693 G694 R695 G696 C697 I699 R702 I705 | S577  second: N701 H645 S643 H646 G648 T651 V650 R647 L652 Q644 |
| MS00464 | 1SQ0_A | L512 K549 W550 D560 G561 S562 H563 A564 Y565 R571 K572 R579 I580 E596 K599 Y600 F603 Q604 S607 K608 D610 R611 E613 R632 N633 R636 P702 | null |
| MS00465 | 1SQ0_A | L512 K549 W550 D560 G561 S562 H563 A564 Y565 R571 K572 R579 I580 E596 K599 Y600 F603 Q604 S607 K608 D610 R611 E613 R632 N633 R636 P702 | E700 A698 C695 C509 L697 D696 |
| MS00671 | 1D4V_B | T129 R130 G131 R132 E155 R158 S159 G160 H161 Y189 R191 F192 Q193 E195 E236 Y237 L239 D269 H270 | N202 S232 C230 W231 |
| MS00742 | 1G9M_H/L | L:W94 P95  H:I52 I54 L55 V57 H59 E103 A104 E106 G107 E108 Y109 R110 N111 | H: P62 Y60 A61 H63 W47  L: best:L2 P95 N92 P96 Q89 Y49 R97 T99 Y98 N93 Y91 W94  second: H200 P206 P115 |
| MS00976 | 3BT1_A | E207 N208 L209 P210 Q211 N212 G213 R214 Q215 G239 P240 N242 T289 S291 | Length restriction |
| MS00984 | 1EER_A | L5 D8 S9 R10 V11 E13 R14 Y15 L16 L17 K20 T44 K45 V46 N47 F48 Y49 K52 Q78 D96 K97 V99 S100 R103 S104 T107 L108 R110 R131 I133 K140 R143 V144 N147 R150 G151 K154 L155 | C161 R162 |
| MS01004 | 1MQ8_B | M140 S141 Q143 P144 T175 L204 L205 T206 N207 E241 A242 T243 D244 S245 K263 H264 S270 | null |
| MS01036 | 3EZE_B | N312 L314 H315 T316 R317 A319 A320 Q321 V323 K324 K327 K340 K345 S346 L347 F348 K349 L350 Q351 T352 L353 G354 L355 T356 E385 | T316 L314 T359 T356 H315 R317 L355  second: L380 K379 H376 |
| MS01037 | 3EZE_B | N312 L314 H315 T316 R317 A319 A320 Q321 V323 K324 K327 K340 K345 S346 L347 F348 K349 L350 Q351 T352 L353 G354 L355 T356 E385 | null |
| MS01038 | 3EZE_B | N312 L314 H315 T316 R317 A319 A320 Q321 V323 K324 K327 K340 K345 S346 L347 F348 K349 L350 Q351 T352 L353 G354 L355 T356 E385 | Length restriction |
| MS01061 | 1MQ8_B | M140 S141 Q143 P144 T175 L204 L205 T206 N207 E241 A242 T243 D244 S245 K263 H264 S270 | Q303 K305 E301 L298 I306 K304 L302 C299 L295 N163 R189 K296  T300 S162 D297 K159 I288 K160 K190 K294 |
| MS01062 | 1MQ8_B | M140 S141 Q143 P144 T175 L204 L205 T206 N207 E241 A242 T243 D244 S245 K263 H264 S270 | Length restriction |
| MS01063 | 1MQ8_B | M140 S141 Q143 P144 T175 L204 L205 T206 N207 E241 A242 T243 D244 S245 K263 H264 S270 | Length restriction |
| MS01105,10,15 | 1II4_A | F21 P22 P23 G24 H25 F26 K27 K30 Y33 K35 G37 G38 F40 R53 Q63 L64 Q65 A66 E67 E68 R69 G70 V72 S73 Y82 V97 E105 R106 L107 N110 N111 Y112 N113 K144 L149 P150 M151 | L149 S152 P29 D57 P150 M151 L64  second: P45 S78 V77 |
| MS01154 | 1HX1_A | T45 D46 N57 A60 M61 N62 N65 R258 R261 R262 T265 A266 R269 S281 E283 I284 D285 S286 G290 D292 Y294 | T222 S221 |
| MS01190 | 1G1S_D | E606 L608 D609 F612 L613 P614 T616 | Length restriction |
| MS01191 | 1G1S_D | E606 L608 D609 F612 L613 P614 T616 | Length restriction |
| MS01192 | 1G1S_D | E606 L608 D609 F612 L613 P614 T616 | Length restriction |
